# Eco-Evolutionary Feedback and the Tuning of Proto-Developmental Life Cycles

**DOI:** 10.1371/journal.pone.0082274

**Published:** 2013-12-18

**Authors:** Eric Libby, Paul B. Rainey

**Affiliations:** 1 New Zealand Institute for Advanced Study, Massey University, Auckland, New Zealand; 2 Santa Fe Institute, Santa Fe, New Mexico, United States of America; 3 Max Planck Institute for Evolutionary Biology, Plön, Germany; University of Illinois, United States of America

## Abstract

Multicellular organisms depend on developmental programs to coordinate growth and differentiation from single cells, but the origins of development are unclear. A possible starting point is stochastic phenotypic variation generated by molecular noise. Given appropriate environmental conditions, noise-driven differentiation could conceivably evolve so as to come under regulatory control; however, abiotic conditions are likely to be restrictive. Drawing from an experimental system, we present a model in which environmental fluctuations are coupled to population growth. We show that this coupling generates stable selection for a single optimal strategy that is largely insensitive to environmental conditions, including the number of competitors, carrying capacity of the environment, difference in growth rates among phenotypic variants, and population density. We argue that this optimal strategy establishes stabilizing conditions likely to improve the quality and reliability of information experienced by evolving organisms, thus increasing opportunity for the evolutionary emergence of developmental programs.

## Introduction

The emergence of multicellular organisms from single cells marks a major transition in evolution [1]. Of prime importance is the evolution of mechanisms that integrate and align the activities of single cells with those of the collective [2]–[6]. In extant multicellular organisms such integration is brought about by developmental programs which regulate cellular activities across space and time – including those necessary for the formation of a multicellular body from a single cell propagule [2], [5], [7]–[9]. By virtue of such developmental programs multicellular organisms effect their own reproduction. Indeed, the earliest multicellular developmental programs were likely those that determined the reproduction of groups, thereby ensuring that newly emergent groups became units of selection [4]. Although developmental programs are pervasive in the multicellular world, their evolutionary origins are unclear [2], [5], [9]–[11].

The most primitive developmental programs are likely to have been rudimentary and based on little other than noise [8]–[10], [12]. For example, a mutation, or an abrupt shift in environmental conditions, might cause a cell of one specific genotype to stochastically switch between two or more phenotypes [13]. If cells of different phenotypes are selectively favored, then the capacity to switch is likely to become established within a population [14]–[20]. Subsequent modification might even lead to the evolution of regulatory mechanisms that tune the production of cell types to specific environmental cues [8], [10], [13], [14], [21].

Molecular details by which stochastic mechanisms of phenotype switching might come under regulatory control are unknown [13]. Specific genetic events are clearly required. However, their fixation requires appropriate opportunity. Such opportunity is likely to depend on environmental factors that maintain stochastic switches for sufficient lengths of time to allow for eventual accommodation within regulatory networks.

Fluctuating environments are known for their ability to select for – and maintain – stochastic mechanisms of gene expression [15], [16], [18]–[20]. While in principle such environments are sufficient to provide opportunity for stabilization of stochastic switches, fluctuating conditions wrought by external factors are unlikely to oscillate in a constant manner. Without regular oscillations, the rate of switching is likely to be subject to change. However, if there is feedback between organism and environment, then stabilization may be possible. In this regard, ecological circumstances that couple environmental change to population growth may have been central to the emergence of primitive developmental programs.

By coupling environmental change to population growth, organisms exert some control over the selective pressures they face [22]–[25]. For example, organisms that reproduce quickly can, as a consequence of exponential growth, reach large population sizes rapidly. If there is coupling between population growth and environmental change, then this will provoke environmental change on a similar time scale. Such rapid environmental change is likely to increase the selective pressure on populations to switch phenotype. Organisms that reproduce slowly, however, are likely to reduce the rate of environmental change and thus relieve selection for rapid switching. At the same time, organisms that reproduce slowly risk being outcompeted by mutants with the capacity for rapid reproduction. Because the activity of an organism influences the selective pressures it experiences, organismal success is determined by both individual reproduction and switch rates, as well as by the reproduction and switch rates of competitors. As populations evolve, so too does the frequency of environmental oscillations. This adds complexity because optimal switching rates may need to be tuned to particular population compositions and sizes.

**Figure 1:**
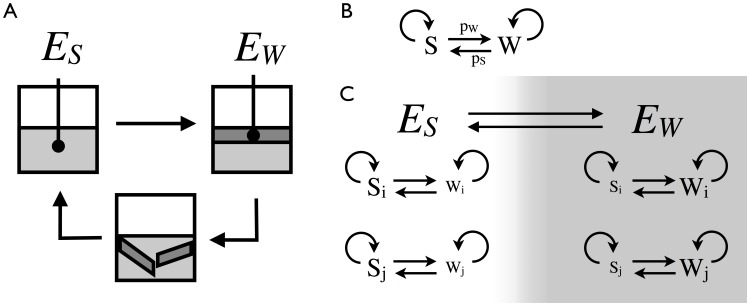
Model schematics. (A) The upper left (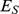) represents initial growth in an oxygen rich broth that favors the smooth 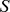 phenotype. As organisms grow and divide, they consume oxygen and eventually the air-liquid interface is the only place with available oxygen. This environment (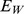) selects for the wrinkly 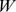 phenotype because it forms a mat at the air-liquid interface through production of a cell-to-cell glue. The mat grows and ultimately collapses under its weight, causing oxygen to flood back into the broth. This, in a sense, returns the system to its starting point. (B) 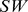 organisms are modeled as stochastic switchers whose one genotype is capable of giving rise to either an 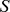 type or a 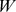 type at division. The 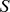 type produces a 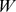 type with probability 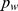 and the 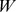 type produces an 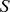 type with probability 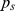. (C) Two organisms 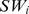 and 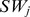 compete in environments 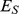 and 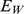. Each organism stochastically switches between 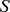 and 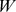 phenotypes which are suited to environments 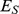 and 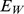, respectively. The environments switch when the total population reaches a fixed value, the carrying capacity 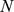, analogous to the amount of oxygen in the broth or maximum weight supported by a mat.

Here, we consider a biological model for this coupling based on experimental populations of the bacterium *Pseudomonas fluorescens* [11], [26]–[30]. When grown in a simple laboratory microcosm *P. fluorescens* establishes a cycle of environmental states and phenotypes [26], [30] (Figure 1a) that produces a primitive life cycle by which groups of cells leave group-level offspring via a propagule stage [11]. In this way, *P. fluorescens* also serves as a model for studying the evolutionary origins of multicellularity. Using a combination of mathematical models and simulations we show that the coupling of environmental change to population growth selects for cells that switch between stages of the life cycle at high frequency. The high rate of switching favors types that switch via an epigenetic mechanism over those relying on mutation. Our models also show that there is an optimal switching strategy which is largely independent of environmental carrying capacity, the number of competitors, the switching strategies of competitors, and the relative differences in growth rates of different types. Switching types that adopt this optimal strategy can dominate a variety of environmental conditions. Importantly, this optimal strategy – arising from the coupling of phenotypic and environmental states – stands to generate stabilizing conditions likely to improve the quality and reliability of information experienced by the evolving organism, thus increasing opportunity for the evolution of developmental programs.

### Models

We consider an environment that switches between two states: 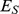 and 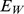. Switching is not stochastic, but rather, is driven by organisms in the environment. The environment is populated by 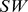 organisms, that is, organisms with the capacity to stochastically switch between one of two phenotypes: 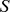 or 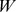. Organisms expressing the 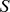 phenotype thrive in 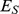 but not in 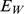, conversely those with the 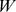 phenotype grow well in 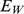 but not in 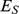. The population expansion of each 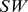 organism in a given environment is governed by four parameters: growth rate of the 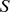 types (“growth” is used in the sense of population growth, i.e. reproduction), growth rate of the 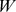 types, probability that an 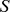 type produces a 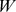 type on division, and the probability that a 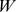 type produces an 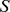 type on division (Figure 1b).

The environment switches states when the total population of 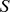 and 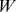 types reaches a fixed value 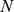. 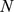 might represent the carrying capacity or the total number of cell divisions permitted by the nutrient content of an environmental state. By this measure, environmental transitions depend on the absolute populations of the 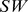 organisms and not the relative abundances of types. It is not necessary that the 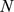 value for the 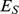 to 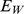 transition be the same as the 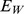 to 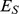 transition.

We represent the expected population of a particular 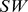 genotype growing in an environmental state, say 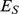, by a set of finite difference equations (Eqn Set 2) that specify the abundance of each phenotype (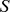 and 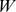). These are similar to equations of stochastic switchers and life cycles elsewhere [31], [32]. The step size 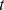 is defined in terms of cell divisions of the faster growing phenotype: 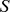 types when in 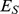 and 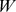 types when in 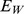. By defining the step size in terms of cell divisions, the number of parameters is reduced by one. The transitions between types are governed by 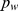, the probability an 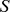 type gives rise at division to a 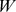 type, and 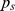, the probability a 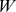 type produces an 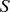 type. Since 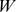 types do not reproduce as quickly as 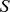 types in 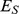, we scale their growth rate by 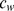 (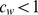) which is the average fraction of 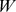 types that grow per cell division of 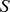 types, that is, the population’s geometric mean per step.
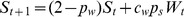
(1)

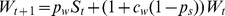



For population growth in 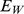, the equations take a similar form. Here, the transition probabilities 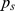 and 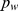 become 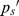 and 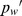 to indicate that the probability of one phenotype producing another may be environmentally dependent. The 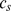 term is the analog of 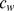 and corresponds to the slower reproduction of 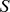 types in 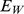.
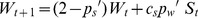
(2)

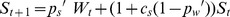



We simulate competition within an environmental state by iterating these equations and computing the population size of each type (see File S1 for computer code). The finite difference equations enable rapid calculations using matrix multiplication in the numerical software MATLAB(version 7.12.0.635 Natick, Massachusetts: The MathWorks Inc., 2011). Often populations reached 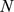 between two rounds of discrete division, say 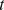 and 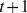. We determine the number of new types to add to the populations at time 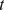 by proportionally scaling the number generated between 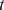 and 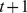 which is the finite approximation to growth limitations imposed by a carrying capacity in Verhulst differential equations.

## Results

Drawing inspiration from experimental populations of *Pseudomonas fluorescens*, we couple environmental oscillations to populations of 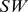 organisms which stochastically switch between two phenotypes 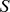 and 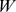 (See Models and Figure 1c). This coupling ties the success of an organism to the dynamics and composition of the population. In actual biological systems there may be hundreds of 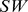 genotypes with different phenotypic characteristics competing simultaneously. Since the dynamics of each genotype in our model is governed by 6 parameters (See Models and Figure 1b & c), the system can become intractable. As a consequence, we use a bottom-up approach in which we analyze a reduced system and then add successive layers of complexity to determine how they contribute to the full model.

### Reduced System

We begin by considering competition between two 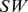 organisms in environment 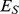. Populations of both organisms are founded by a single 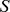 type with growth continuing until the total population reaches 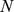, at which point the environment switches to 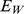. Since 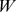 types are more suited to 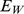, the organism with the most 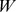 types at the time the environment transitions from 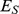 to 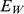 has an advantage. For the moment, we do not consider growth in the 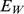 environmental state and are, therefore, interested in strategies that will best prepare an organism for the next environmental state. We further assume that 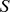 types of each 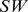 organism are equally fit and the 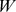 types do not grow at all. Both of these assumptions will be lifted later but in this limited case, the only difference between competitors is the probability (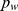) an 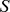 type produces a 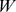 type.

**Figure 2:**
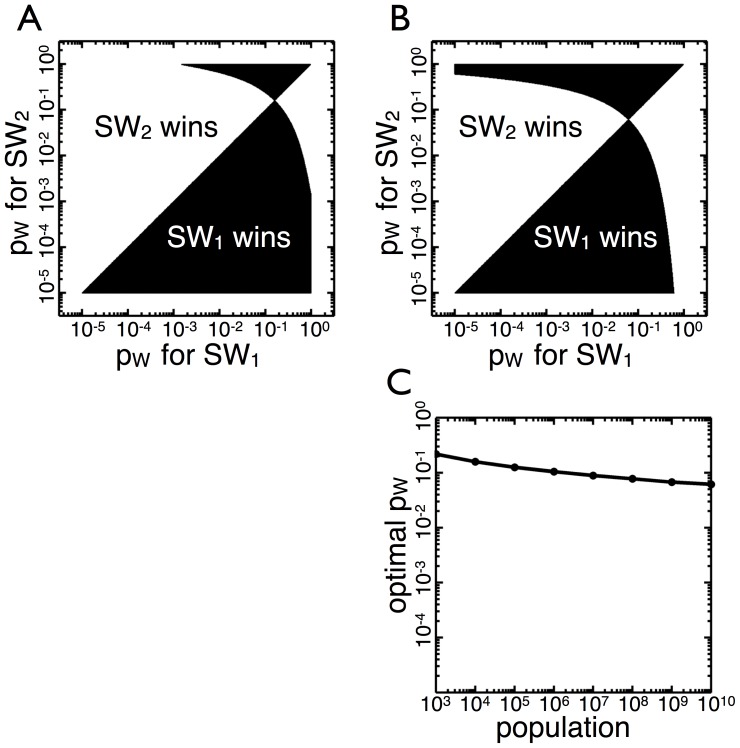
Competition between 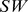 genotypes with different transition probabilities, 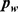. (A) The black area corresponds to specific combination of 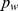 values for two 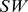 genotypes in which 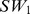 leaves more 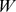 types than 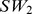 when the environmental state changes to 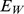 at 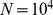. The white area represents the converse. There is an optimal 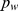 (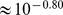) which results in more 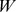 types compared to any other 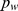. (B) The same as A but with 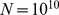. The optimal 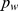 has decreased to 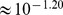. (C) The optimal 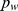 is calculated for 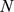 values between 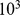 and 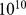. As 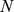 increases over seven orders of magnitude, 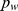 remains relatively constant, decreasing less than an order of magnitude.

To determine which strategy for 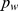 leaves the greatest number of types adapted to the next environmental state, we systematically compete pairs of 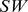 organisms with different, fixed values of 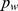 (ranging from 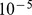 to 1). For each competition, we compute the number of 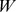 types produced by each organism during growth in 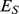 before the environmental state changes to 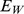, that is, the point at which the total population reaches a fixed number 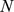 (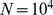 in Figure 2a). For values of 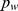 below 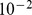, the organism with the higher transition probability (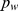) leaves more 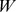, i.e. it “wins” because it sets up a numerical advantage in the next environmental state. At high 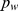 values (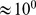), the opposite is true, such that the organism with the lower 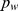 wins. This occurs because organisms with lower 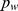 initially invest more in the fast-growing 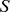 type and, thereby, defer the expected production of 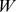 types until they have amassed a large population of 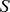 (they thus contribute many more 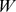 types). The result is a single optimal strategy.

Qualitative outcomes of the competition remain unchanged even when the population size necessary to effect an environment switch is as large as 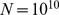 (Figure 2b). There remains a single optimal strategy which can beat higher and lower transition probabilities. The higher carrying capacity (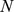) enlarges the range (top shaded region) in which organisms with high transition probabilities can be defeated by those with low transition probabilities. Because higher 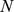 means populations go through more rounds of cell division, the production of 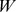 types can be deferred until later rounds of division. Consequently, higher population sizes 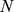 lead to a slight reduction in the optimal transition probability from 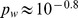 in 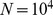 to 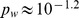 in 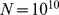.

To see how the value of 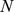 affects the optimal 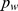, we vary 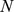 from 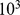 to 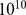 and calculate the optimal 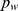 (Figure 2c). As 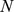 increases the optimal 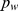 decreases monotonically approximately half an order of magnitude, but this change is small in comparison to the seven order of magnitude difference in 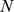 . Thus, the optimal transition probability remains close to 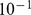 for a wide range of population sizes and is, therefore, insensitive to environmental carrying capacity.

### Unfair Competitions

Thus far, the competition between the two 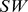 organisms has been fair with each starting at the same initial number and with the same growth rate. In actual biological systems, new competitors can invade or emerge via mutation. This is likely to result in an unfair competition in which one competitor faces an initial numerical or growth rate disadvantage. Disadvantages can also occur when an organism trades reduced growth rate in one environment against heightened performance in a second. Such an organism has a growth disadvantage in one environmental state and an initial numerical disadvantage in the second. It is likely that by changing transition probabilities organisms may compensate for any such disadvantage: a slower reproducing organism may do better if it has a higher transition probability. If compensation occurs then organisms evolving in such ecological conditions will face different selective pressures depending on their frequencies in the population.

**Figure 3:**
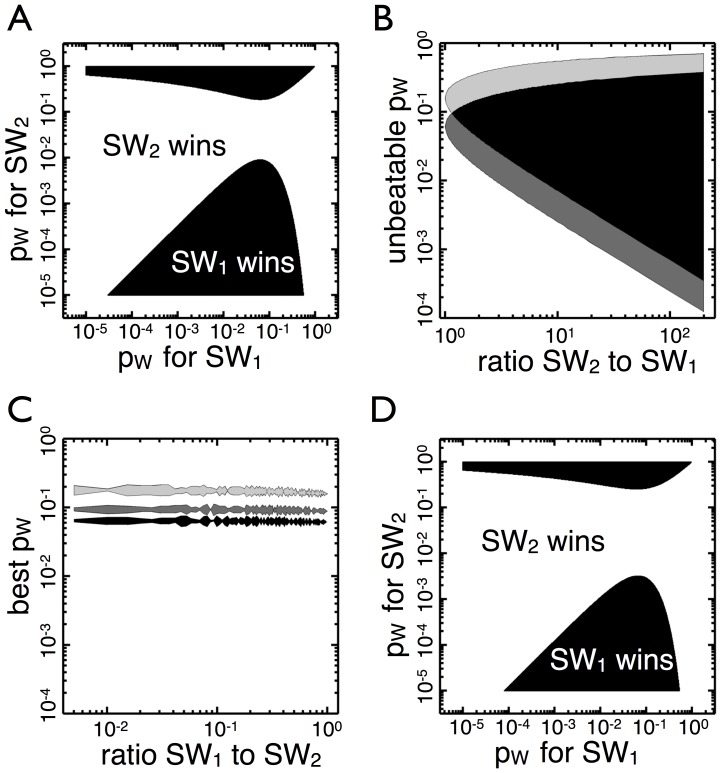
Competitions in which one organism has a numerical or growth advantage. (A) 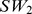 begins growth in 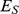 with a 3: 1 advantage to 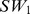 (150: 50, growing to N = 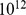). The black area corresponds to combinations of 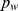 in which 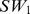 leaves more 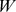 types than 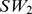 (white is the converse). The gap between the two areas corresponds to a range of transition probabilities in which 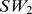 maintains an advantage independent of the transition probability of 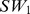. (B) The range of transition probabilities for which 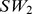 maintains an advantage is shown as a function of the initial numbers advantage. The light gray area corresponds to 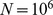, the dark gray is 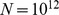, and black is the overlap. Increased carrying capacity (N) shifts the range of transition probabilities down but does not greatly alter the total area. (C) The transition probability 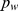 that minimizes the losses for 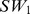 is shown as a function of its numerical disadvantage. The light gray is 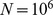, the dark gray is 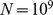, and the black is 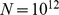. There is a slight increase in the optimal transition probability as the disadvantage increases (left of the graph) but it is less than a tenth of an order of magnitude away from the optimal transition probability when the competition is fair. (D) 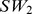 has a growth advantage compared to 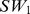, dividing 10% faster. The two begin with one organism and divide until 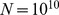. Again 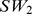 has a range of unbeatable transition probabilities, and the graph resembles A.

To see how an initial numerical advantage affects the competition, we allot one organism, 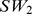, a three fold advantage (150: 50) over the other, 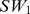, and allow the total population to increase until the environmental state changes at 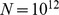 (population expansion from 2 to 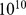 is the same fold as growing from 200 to 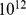). The initial numerical advantage allows 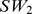 to maintain an advantage for a range of transition probabilities (Figure 3a), including the transition probability that is optimal in a fair competition. Still, 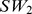 can be overtaken if its transition probability is below 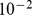 or if it is too high. For larger numerical advantages the range of unbeatable transition probabilities increases (Figure 3b). With a 100 fold advantage, an organism can maintain a numerical advantage for transition probabilities spanning three orders of magnitude. The value of 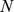 shifts the range of unbeatable transition probabilities such that lower 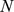
’s require higher transition probabilities to maintain the advantage while higher 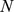
’s permit lower transition probabilities.

If an organism with an initial numerical disadvantage has the transition probability that is optimal in a fair competition then it minimize its chances of remaining outnumbered in the next environment (Figure 3c), regardless of the size of its disadvantage. Thus, the optimal transition probability in fair competitions is also the best in unfair competitions– whether an organism has an initial numerical advantage or disadvantage.

An initial numerical advantage is qualitatively similar to a growth advantage. The rate at which a homogeneous 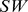 population grows depends not only on the doubling times of both phenotypes, but also the corresponding transition probabilities. For example, an 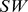 population growing in 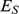 that never produces the slow-growing 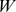 types will reach higher numbers than one that frequently produces 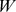 types. Instead of comparing absolute population growth rates of 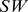 genotypes, we compare the growth rates of only the 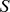 types in the 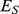 environment. As in the case where one genotype has an initial numerical advantage, a genotype with a faster growth rate (10% increase) is afforded a range of unbeatable transition probabilities (Figure 3d).

### Growth Disparity between Phenotypes

**Figure 4:**
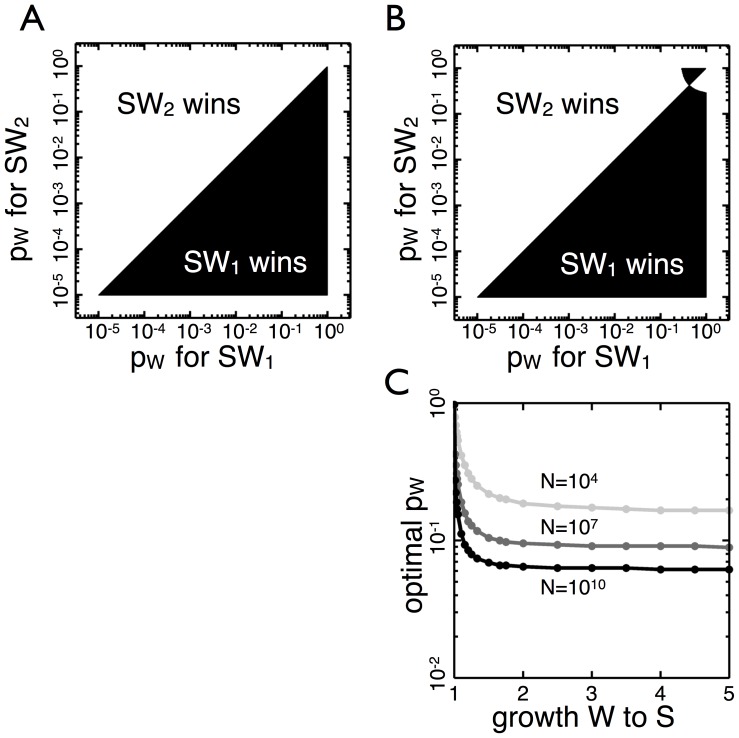
The effect of the maladapted type’s growth on the optimal transition probability. (A) Two 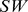 organisms compete (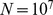) with different transition probabilities, 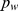, when 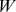 types grow at the same rate as 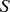 types– when the cost of maladapted type (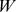) is minimal. Without a penalty for producing 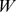 types the organism with the higher 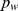 always wins. (B) 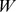 types grow 1% slower than *S* types (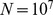). The optimal transition probability decreases and the top black area of the transition probability competition graph increases. (C) The optimal 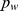 is shown as a function of the growth discrepancy between *S* and *W* types for three different values of *N*. The optimal transition probability when *W* types grow by a factor of 1.5 times slower than *S* types is approximately the same as when the *W* types do not reproduce at all.

A disparity in growth rates between 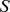 and 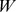 phenotypes creates a cost to producing the slower dividing, maladapted type (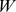 in 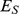 or 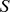 in 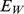). For each maladapted type that is produced there is less investment in the faster growing phenotype, and consequently the average growth rate for the 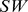 genotype decreases. Thus far, we have considered the case in which the maladapted types impose a maximum cost because they never divide. At the opposite extreme is the minimum cost in which 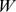 types grow at an identical rate to 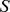 types in 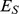 (Figure 4a). Since the goal is to leave the most 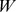 for the next environmental state (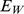) and there is no growth disadvantage to the 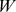 types, the optimal transition probability is 1, 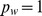.

If a small cost is imposed so that the growth rate of *W* is reduced by 1–5% then the optimal transition probability drops super linearly (Figure 4b). Further increases in the cost show that the optimal transition probability quickly drops close to 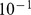 (Figure 4c). As the growth rate of the *W* types approaches half that of the *S* types, the optimal transition probability is the same as when the *W* types do not grow at all. The insensitivity of the optimal transition probability to the growth rate of the maladapted type is independent of *N*, the population size at which the environment transitions. Thus, as long as there is a growth disparity between the two phenotypes, there is selective pressure to stably produce both types at high frequency.

### Closing the Life Cycle

Since we have only been considering one environmental transition 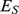 to 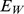, we have focused attention on the probability of producing *W* types from *S* types. Indeed, this determines the preparedness of an organism to succeed when the environment switches to the 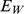 state. To survive multiple environmental oscillations, however, organisms must be able to switch back and forth between types. The probability that a *W* type produces an *S* type upon division (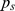) is therefore essential to surviving the transition from 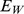 back to 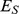. While essential during growth in 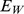, 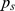 counteracts the effect of 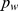 during growth in 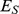. Since the success of *SW* organisms depends on both transition probabilities, 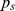 and 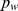, we investigate their interactions and the effects on the optimal strategy.

**Figure 5:**
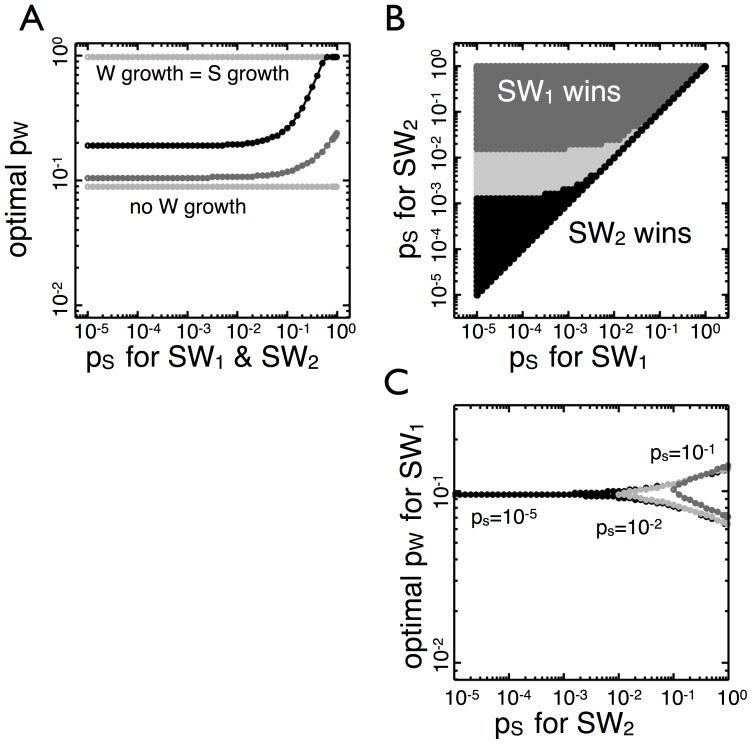
The effect of the probability 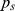 on the optimal transition probability. (A) The optimal value of 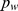 is shown as a function of a fixed 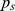 for both organisms. The light gray horizontal lines represent the trivial cases when *W* types never divide (bottom) or grow at the same rate as *S* types (top). When *W* grows 90% of the rate of *S* (black) or 66% of the rate of *S* (dark gray) the optimal 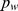 does not change until 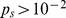, at which point it increases to counteract 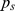. (B) For competitions between *SW* organisms with different values of 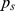 (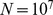), the organism with the lower 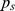 has an advantage and has a range of transition probabilities for which it does not lose in frequency. The shading color corresponds to the size of this range: 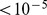 (black), 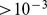 (light gray), and 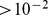 (dark gray). For larger differences in 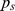, there exist more transition probabilities 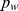 that an organism can adopt to maintain or gain in frequency. (C) The optimal 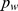 is shown as a function of the advantage in 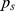 for competitions from B. The 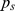 for 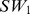 is fixed at either 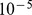 (black), 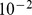 (light gray), or 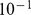 (dark gray). The range of the optimal 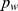 (area between curves of the same color) is narrow until the 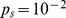 for 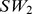. Thus, the range depends on the magnitude of the advantage only when 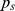 is sufficiently high.

To uncover how 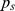 and 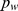 interact, we first fix 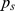 for both *SW* organisms to ensure that their *W* types have the same probability of producing *S* upon cell division. For different combinations of 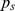 and growth rates of *W*, we calculate the optimal transition probability 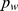 (Figure 5a). The upper and lower bounds of 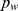 are set by the maximal and minimal cost of the maladapted phenotype, that is, the upper bound is at the point where *W* never divides and the lower bound is defined by the point at which *W* divides at the same rate as *S*. The optimal 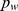 in either case does not depend on the value of 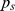. When *W* never divides, 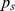 has no effect on the optimal 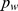 because *W* never has the opportunity to produce *S*. When *W* divides at the same rate as *S*, the optimal strategy is to produce *W* types as often as possible regardless of how often *W* types give rise to *S* types. For intermediate growth rates, when *W* grows at 66% or 90% the rate of *S*, the optimal value of 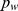 is unaffected by 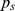 as long as 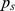 is low (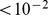). Once 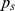 increases above 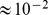 the optimal 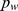 also increases. Since the effect of 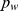 is to produce *W* types, high values of 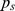 counteract this effect and thereby act as a leak. In order for an organism to leave enough *W* types at the end of growth in an environmental state, it must increase 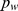 to offset the loss of potential *W* types due to 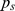.

When the condition that competing *SW* organisms have identical values of 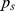 is relaxed, the organism with the lower 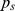 is more successful (Figure 5b). If there is a large discrepancy in 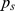, the organism with the lower 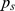 has a range of unbeatable 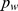 transition probabilities similar to what was observed in the case of unfair competitions (Figure 3). High values of 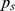 are detrimental because they impede accumulation of *W* types. During growth in 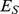, the best strategy is to balance the faster division of *S* types with the production of *W* types that are essential to future success. Each time a *W* type gives rise to an *S* type it results in one *W* and one *S*. This same result could be obtained more quickly if an *S* type divides to give rise to a *W*. The delay due to waiting for a *W* type to divide as opposed to an *S* type, effectively reduces the growth rate of the *SW* genotype and allows those with lower values of 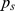 to dominate.

Although the organism with the lower 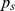 has an advantage it is unclear how this affects the optimal transition probability 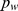. We assign organism 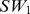 a lower back probability, 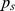, than its competitor 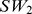 and calculate the range of unbeatable transition probabilities 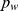 for 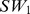 (Figure 5c). If 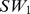 has 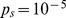 there is a narrow range of optimal transition probabilities 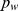 in which winning is possible even when 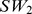 has a 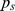 value two orders of magnitude higher. By contrast if 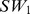 has a 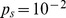 and competes against an 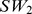 whose 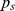 is only slightly higher then there is a wide range of unbeatable transition probabilities – as wide as if the 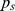 for 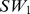 had been 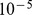. This reflects the fact that 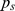 does not affect the optimal strategy unless it is sufficiently frequent. Furthermore the transition probability 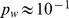 remains unbeatable regardless of the advantage in 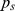.

### The Number of Competitors

As organisms grow and colonize environments, it is likely there will be more than just two competing genotypes. With more competitors, there is more opportunity for complex interactions between diverse strategies. It is possible that the previous optimal transition probabilities do not fare well against combinations of strategies. Furthermore, the competition may no longer produce a single unbeatable strategy. To explore these possibilities, we consider an expanded competition between hundreds of genotypes.

Increasing the number of competitors, however, means that it is no longer feasible to systematically search parameter combinations as was done before. Instead, we establish a tournament to find the best strategy in the space of transition probabilities. First, we randomly generate competing genotypes by sampling transition probabilities from a uniform distribution in log space (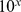, where 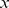 is sampled from a uniform distribution between 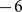 and 0). Each genotype is defined by its four transition probabilities: 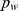 in 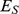, 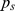 in 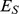, 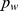 in 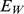, and 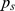 in 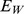. We then simulate the population dynamics of competing genotypes through a full environmental cycle: beginning in 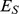, moving to 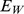, and returning back to 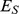. In each environmental state the population grows by a factor of 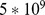, which is equivalent to two organisms growing to a population of 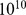. Maladapted types (*W* in 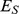 and *S* in 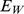) require twice the time to reproduce as the adapted types (*S* in 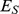 and *W* in 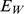). As the environment switches states only the previously maladapted type can pass through, e.g. when 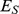 changes to 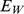 only *W* types can pass through. After one complete cycle the most abundant genotype is the winner and allowed to continue to the next round. The remaining competitors are replaced with new competitors whose transition probabilities are identical to that of the winner, except that one is randomly changed. This process allows tuning of the winning strategy as each new winner represents the adjustment of one parameter. If all transition probabilities were changed simultaneously it would take longer to find the optimal strategy because optimal parameters would be linked with potentially poor parameters.

Due to the symmetry of the system, 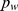 in 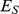 should act similarly to 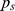 in 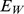 because both of these probabilities correspond to production of the type better suited to the next environment. From our analysis of the reduced model, we predict a successful organism will have these probabilities tuned close to 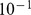. Likewise, the other two transition probabilities (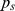 in 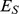 and 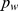 in 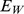) share a similar role in opposing the direction of environmental change. Tuning of these probabilities should result in as low a value as possible (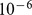 in this expanded model).

**Figure 6:**
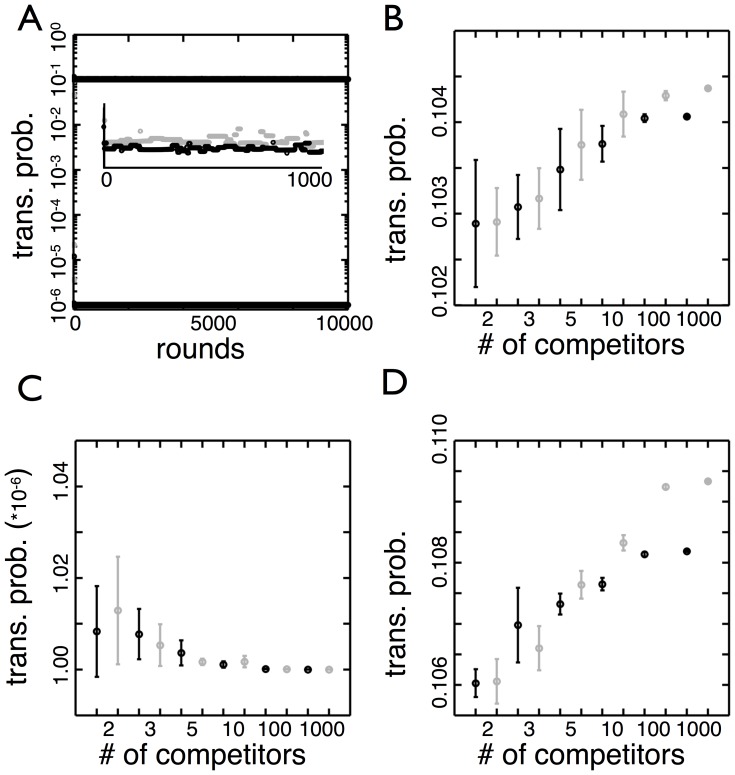
Competition between more than two genotypes. (A) The transition probabilities of the winning genotype are shown over 10,000 environmental cycles (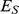 to 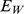 to 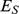). Each cycle begins with 1,000 genotypes in equal number and at the end of a full cycle the most abundant genotype (the winner) advances to the next round to compete against new, randomly generated genotypes. The transition probabilities of the winner are grouped by environmental state: black is 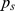 and 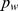 in 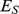 and gray is 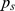 and 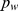 in 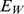. The inset is a magnification that shows the winner changes frequently but in the long run the winner appears as unbroken lines. (B) The transition probabilities (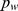 in 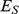 is black and 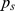 in 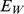 is gray) of the winning genotypes are shown as a function of the numbers of competitors. Each point is the average of 10 runs of tournaments (as shown in A) sampled every 100 rounds after 2500 rounds (“burn-in phase”). The error bars are the standard deviation of the 10 runs. The values remain close to 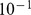 for different numbers of competitors. (C) The same as B but this shows the other two transition probabilities (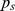 in 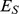 is black and 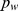 in 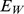 is gray) for winning genotypes. The values are all close to the lower bound of transition probabilities considered (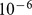), so the number of competitors has no effect. (D) The transition probabilities of the winning genotype are shown when the probabilities do not depend on the environmental state. Due to the symmetry of the system the optimal 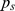 (gray) and 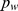 (black) are identical. There is a statistically significant increase in the transition probabilities for the competition of 1000 competitors versus 2, but the magnitude of difference is small.

For numbers of competitors ranging from 2 to 1000, we conduct the tournament for 10,000 rounds 10 independent times (see File S1 for sample computer code). An example simulation using 1,000 competitors (Figure 6a) shows that though there are frequent replacements, the winning transition probabilities are close to the predicted values. The optimal values for 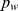 in 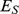 and 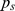 in 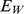 (black and gray in Figure 6b) are between 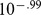 and 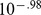 for all competitions between 2 to 1000 competitors. While the optimal transition probabilities for tournaments with 1,000 competitors are higher (significance tested with t-test, 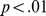) than those with 2 competitors, the relative difference is less than 2%. The optimal values for 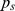 in 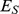 and 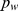 in 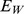 (black and gray in Figure 6c) also match with predictions and are close to 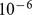.

So far, the transition probabilities between phenotypes have varied with environmental state but this may not always be the case. If, instead, the probabilities are independent of the environment, then only two parameters define a genotype in this tournament. Moreover, this means while 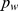 is beneficial in 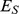 it is a hinderance in 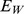. To see how this affects the optimal strategy, we repeat the tournaments with transition probabilities that do not depend on the environment (Figure 6d) and find that both transition probabilities are close to 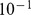 regardless of the number of competitors. This indicates that the benefit for a high 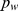 in 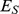 outweighs its cost in 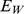. As found previously, the transition probabilities increase as the number of competitors increases (significance tested with t-test 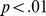) but the difference is small in comparison with the change due to the number of competitors.

### Exclusions during Environmental Transitions

As environmental states alternate in the *Pseudomonas fluorescens* experimental system, phenotypes are excluded so that only one type can advance to the next state. As oxygen in the broth becomes exhausted by growth of *S* types, opportunity is created for mat-forming *W* types that colonize the air-liquid interface. Once a mat collapses and oxygen penetrates into the broth phase, only the unentangled *S* types are free to grow. This exclusion, as incorporated into our model, is perfect in that none of the other type is able to pass through to the next environmental state. In other biological systems, it is possible that fractions of both types might survive changes in environmental states, i.e. the environmental transitions do not perfectly filter phenotypes. Such imperfect filtering may reduce the pressure to switch at high frequencies and, thereby, reduce the effectiveness of the switching strategy that was optimal under perfect filtering.

**Figure 7:**
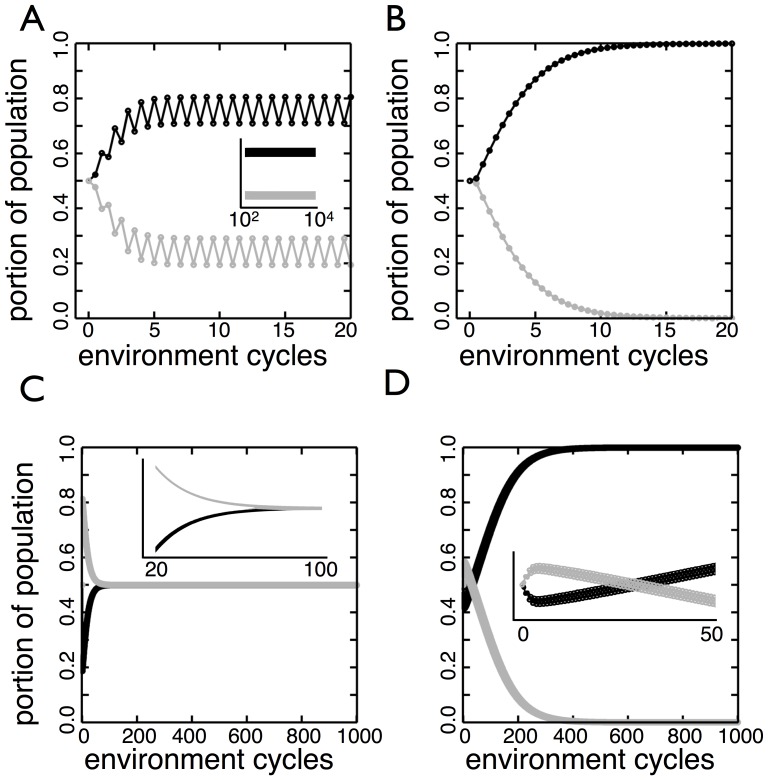
Different behaviors with an imperfect filter between environments. (A) The populations of 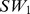 (black) and 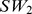 (gray) form a stable oscillation as the environments cycle between 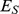 and 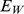. The transition probabilities are presented 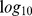 in the order of 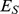 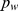, 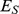 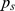, 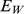 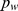, and 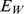 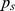. For 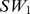 the transition probabilities are 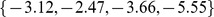 and for 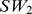 they are 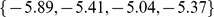. (B) 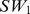 (black) drives 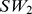 (gray) extinct. For 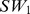 the transition probabilities are the optimal set using perfect filters 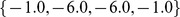 and for 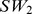 they are 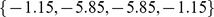. (C) 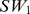 (black) and 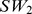 (gray) monotonically approach the same frequency in the population. For 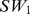 the transition probabilities are 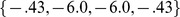 and for 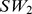 they are the optimal set when perfect filters are used 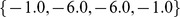. (D) 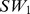 (black) decreases in frequency for five rounds before changing direction and driving 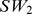 (gray) extinct. The transition probabilities for 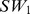 are 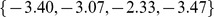 and for 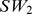 they are 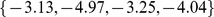.

Imperfect filtering is implemented by allowing a fraction of the type better adapted to the current environment to survive as the environment transitions, for example, some *S* survive as 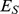 switches to 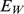. This introduces three new parameters to the model: the fraction of each type that survives along with the total number of types permitted to advance to the next environment (the size of the bottleneck). A full investigation of the effects of these parameters is beyond the scope of this paper. However, we simulate a competition between two organisms through repeated environmental cycles to discover what qualitative dynamics are possible. As the environment transitions from 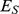 to 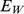 we allow 1% of the *S* types to survive, and likewise for the converse. From a bottleneck size of 10^5^ the populations grow an additional 10^10^ cells, i.e. the carrying capacity is 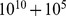. The competing genotypes (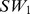 and 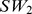) have identical growth rates for *W* and *S* types with maladapted types (*W* in 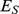 and *S* in 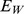) reproducing half as fast as adapted types. The only difference between genotypes is the set of transition probabilities between states. The environments cycle until the populations reach a stable state. The results of the simulations show qualitatively different dynamics depending on the combinations of transition probabilities (Figure 7).

The two types of system dynamics most common to imperfect filters are those involving stable oscillations or extinctions. In a stable oscillation the *SW* organisms cycle between two different frequencies corresponding to growth in the different environments (Figure 7a). Each organism is more adapted to one environmental state and alternates between high and low abundance. In those instances where extinction is the norm (Figure 7b) models show that the approach to extinction can be monotonic or oscillatory depending on whether an organism repeatedly gains in frequency in one or both environmental states.

While stable oscillations and extinctions are common, imperfect filters do permit behavior that was not observed when perfect filters were applied. For example, the switching strategy that was optimal in earlier sections (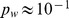, 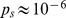 in 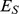 and the converse in 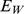) is no longer dominant in the face of an imperfect filter. Figure 7c shows a monotonic approach to a polymorphism in which one organism steadily increases at the expense of the other organism. The decreasing organism has the switching strategy that was previously optimal under perfect exclusion. Although, the steady state frequency could be any value between 0 and 1, we show the case in which both organisms represent exactly half of the population despite their different transition probabilities. Another instance of behavior that is only possible with imperfect filters is the “come from behind win”. In Figure 7d, the gray organism initially increases in frequency over the first five complete environmental cycles and then begins a steady decline to extinction. This example demonstrates that the absolute abundance of an organism is not indicative of how it is performing. The gray organism appears as though it will drive the other organism extinct. With each environmental cycle, however, the organism in black shifts its allocation of types so that at the start of growth in a future environmental state it has the numerical advantage in the fast-growing type. These two examples demonstrate that although coupling environmental transitions to organismal growth stably selects for a switching strategy when perfect exclusion is in operation, it is less straightforward when the environment imperfectly filters types.

## Discussion

Multicellular organisms depend on developmental programs to coordinate their growth and differentiation from single-celled propagules so as to effect a life cycle [3], [5], [7], [9]. Yet, the evolutionary origins of such developmental programs are unknown [2], [5], [9]–[11]. It is likely that early developmental programs began as little more than phenotypic noise in the form of genotypes that probabilistically produce different phenotypes [8]–[10], [12]. Possibly, such phenotypic switching might itself have been the product of evolution in fluctuating or unpredictable environments [14]–[20]. Noisy expression of phenotypes could conceivably come under regulatory control if evolving organisms experience environmental conditions that provide reliable information of appropriate quality [13], [33]. Our models show that if there is coupled feedback between phenotypic and environmental states then this can lead to stabilizing selection for a single optimal switch probability. Circumstances that maintain switches of this kind are likely to define conditions central to the ecology of development.

The findings from our model expand the evolutionary role of phenotypic noise as embodied by stochastic switchers. Typically, phenotypic noise is understood to be an evolved response to uncertain, fluctuating environments: genotypes capable of stochastically switching phenotypes can hedge their evolutionary bets to maximize long-term geometric fitness [34]. Moreover, organisms whose switch rates are better tuned to the frequency of environmental oscillations are fitter. To predict how switching rates evolve requires knowledge of the frequency of environmental fluctuations. Without some force driving regular environmental oscillations, prolonged periods in one environmental state is likely to result in loss of the capacity to switch. In contrast, when environmental oscillations are coupled to organismal growth there is a steady drive to maintain switching. Coupling establishes conditions that select for a single optimal switch rate (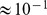 for the type suited to the next environmental state), so organisms can maximize their fitness by adopting this rate regardless of the rates of competing organisms. Furthermore, the fact that this optimal switch rate is high increases selection for organisms to find a non-mutational route to phenotypic innovation. Thus, coupling environmental oscillations to organismal growth produces conditions that favor organisms that tune phenotypic noise towards the optimal switch rate.

While coupling yields a single optimal strategy, it would not be much use if the strategy relied on very specific environmental conditions. For example, if the optimal strategy depended on a particular carrying capacity then small fluctuations to nutrient availability would jeopardize its long-term success. Additionally, if the optimal strategy changed with the composition of the population then there would be frequent pressure to adjust the switching rate. Such sensitivities, however, do not appear in our model. The optimal switching strategy is robust to changes in the carrying capacity of the environment as well as the number, growth rates, and switching strategies of competing organisms. This robustness means that organisms who adopt the optimal strategy can remain dominant despite environmental perturbations. Moreover, there is more opportunity to spread and successfully colonize other environments as long as coupling between phenotypic state and environment is maintained. This extended success affords organisms who adopt the optimal strategy more time to realize a chance mutation that moves phenotypic switching away from noisy mechanisms and towards developmental regulation.

One key assumption of our model is that the probability of producing phenotypes, the switching rates, can evolve. Although stochastic switchers have been engineered to switch at different rates [35], there has not yet been experimental characterization of the capacity of switches to evolve. The degree to which a switch can be tuned through evolutionary processes likely depends on the mechanism of the switch and the underlying regulatory networks [21], [33], [36]. For example, if the switch frequency depends on the binding of a protein to some molecule then the switch might be tuned through modification of the protein’s amino acid composition or its regulation. If, however, the switch were a product of a whole pathway then the number of evolutionary targets would likely increase as would the potential for pleiotropic effects. In either case, it is not clear what switch rates can be obtained or what type of resolution there is in switch rate tuning. Moreover, it is possible that the recurring 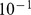 optimal transition probability may be too high for some systems to achieve. In these cases, our model predicts that the highest obtainable transition probability would then be optimal.

Despite the coupling between environmental change and organismal growth in our model, there does not need to be any regularity to the frequency of environmental oscillations– even if the entire population has the same switching rate. This potential disconnect between switch rates and environmental oscillations stems from the way our model implements environmental change. We assume that environmental states do not change until the population of organisms has reached the carrying capacity (*N*). Once the population equals the carrying capacity, the environmental state can change immediately or any time afterwards as long as the population composition does not change before the environmental state changes. As a consequence, the population may evolve to an optimal switching rate without necessarily creating a regular frequency of environmental oscillations. If, however, the environment does change a fixed time after the population reaches *N* then an optimal set of transition probabilities implies the environment will oscillate at a specific frequency.

Although our model captures the basic cyclic ecological conditions present in the *Pseudomonas fluorescens* experimental system, it is unclear how prevalent these conditions are in other biological systems. Certainly there are many instances in which organisms modify the environment to favor another phenotype that they, themselves, produce [22], [25]. This is a particular issue in the evolution of cooperation as cooperative phenotypes modify the environment to favor cheater types who arise via mutations [26], [37]–[39]. Indeed, the *Pseudomonas fluorescens* experimental system has been used to study the evolution of cooperation with the mat formers (W) representing cooperators and the smooth types (S) representing cheats [11]. The key aspect of the experimental system– and our model– is the closed cycle, i.e. how the cheats change the environment to favor the cooperators. The likelihood of this event should determine the prevalence of the ecological conditions. If the cycle can be closed, our model shows that there is a strong pressure to switch rapidly between types.

Finally, the extent to which environmental transitions filter phenotypes turns out to be critical. This makes sense given that the amount of filtering depends in part on the differences between environmental states, i.e., similar environmental states are less likely to filter phenotypes. As environmental states become more alike it is expected that generalist strategies emerge. Conversely, with greater differentiation between environmental states greater phenotypic exclusion is expected, and, therefore, selection for switching at high frequency [28], [29] While it is easier to imagine imperfect filters as being more prevalent in biological systems, perfect filters exist in multicellular organisms in the form of the germ-soma distinction. By denying soma the ability to reproduce without the germ line and vice versa, multicellular organisms use perfect filters to assure the continued reliable production of a life cycle.

## Supporting Information

File S1 **Sample computer code for simulations.**
(PDF)
