# Supplementary material for: Eco-Evolutionary Feedback and the Tuning of Proto-Developmental Life Cycles
Source: PLoS One. 2013 Dec 18;8(12):e82274. doi: 10.1371/journal.pone.0082274 (PMC3867345; doi:10.1371/journal.pone.0082274)
Supplement: File S1 — Sample computer code for simulations. (PDF) [file pone.0082274.s001.pdf]

Code for competitions in Figure 2

```
% Initialization
pwvect=10.^[-5:.01:-.01]; % range of pw values
Nvect=10.^[3:1:10]; % vector of carrying capacity (N)
matres=zeros(length(pwvect).^2,3); % store competition results for an N
matrescell=cell(length(Nvect)); % stores the matres for each N
A=zeros(4,4); % finite difference eqns for # S and W for SW1 and SW2
cw1=0;ps1=0; % W for SW1 doesn't grow, ps1 doesn't matter
A(1,2)=cw1*ps1;
A(2,2)=(1+cw1*(1-ps1));
cw2=0;ps2=0; % W for SW2 doesn't grow, ps2 doesn't matter
A(3,4)=cw2*ps2;
A(4,4)=(1+cw2*(1-ps2));
cs1=1; % growth rate of S is same for SW1 and SW2

% Computation
for i0=1:length(Nvect)
    ct=0; % counter for matres
    curn=1; % Starting point for search of # divisions to reach N
    for i1=1:length(pwvect);
        pw1=pwvect(i1);
        A(1,1)=1+cs1*(1-pw1);
        A(2,1)=cs1*pw1;
        for i2=1:length(pwvect);
            pw2=pwvect(i2);
            A(3,3)=2-pw2;
            A(4,3)=pw2;
            % Search for # of divisions to reach N
            done=0;
            while (done<1)
                B1=A^curn;
                B2=A^(curn+1);
                % initialcond is S1=1 W1=0 S2=1 W2=0
                totpop1=B1(1,1)+B1(2,1)+B1(3,3)+B1(4,3);
                totpop2=B2(1,1)+B2(2,1)+B2(3,3)+B2(4,3);
                if ((totpop1<Nvect(i0)) & (totpop2>Nvect(i0)))
                    diffn=Nvect(i0)-totpop1;
                    newTot1=B1(2,1)+diffn*(B2(2,1)-B1(2,1))/(totpop2-totpop1);
                    newTot2=B1(4,3)+diffn*(B2(4,3)-B1(4,3))/(totpop2-totpop1);
                    ct=ct+1;
                    matres(ct,:)=[pw1 pw2 newTot1/(newTot1+newTot2)];
                    done=2;
```

```
                elseif (totpop2<Nvect(i0))
                    curn=curn+1; % more cell divisions
                elseif (totpop1>Nvect(i0))
                    curn=curn-1; % less cell divisions
                end
            end
        end
    end
    matrescell{i0}=matres;
end

% Plot data for N=10^7
close all
i0=5;% index of Nvect
k1=find(matrescell{i0}(:,3)>.5);
figure;loglog((matrescell{i0}(k1,1)),(matrescell{i0}(k1,2)),'k.');
axis([10^-5.5 10^.5 10^-5.5 10^.5]);
set(gca,'TickLength',[.025 .025],'LineWidth',3,'FontSize',14);
xlabel('SW_1 transition rate','FontSize',14);
ylabel('SW_2 transition rate','FontSize',14);
axis square;
set(gca,'XTick',10.^[-5:1:1]);
```

Code for tournament in Figure 6

```
w2=rng('shuffle'); % Change the randomization

% Properties of the simulation
numofrounds=10000; % number of environmental oscillations
tol=.01; % the discretization size for time, 1/tol = # of steps per unit time

% Properties of the environments
Ns=10^10; % # of divisions that can occur in Es
Nw=10^10; % # of divisions that can occur in Es
maxstartpop=2; % max # of orgs to start in Es or Ew, fractions allowed, all tournaments expand from 2 to 10^10

% Properties of the organisms
startpop=1000; % # of genotypes who begin the simulation.
initdivtime=2; % starting division time for strains
growdisadvfact=2; % fraction of growth by no dominant type


% Data storage for strains
limstrain=startpop; % limit to # of strains possible
strainmat=zeros(limstrain,8); % stores properties of individ. strains
% cols for growth => 1. avg div time | 2. div time in bad env |
i1=1;
strainmat(i1,1:2)=[initdivtime initdivtime*growdisadvfact];
% cols for trans. prob => 3. pSW Es | 4. pWS Es | 5. pSW Ew | 6. pWS Ew |
strainmat(i1,3:6)=10.^(-3*ones(1,4));
strainmat(i1,7:8)=[maxstartpop/startpop 0];
for i1=2:startpop
    strainmat(i1,1:2)=[initdivtime initdivtime*growdisadvfact];
    % cols for trans. prob => 3. pSW Es | 4. pWS Es | 5. pSW Ew | 6. pWS Ew |
    strainmat(i1,3:6)=10.^(-6*rand(1,4));
```

```
    strainmat(i1,7:8)=[maxstartpop/startpop 0];
end
% cols for numbers => 7. num of S | 8. num of W
strainnum=startpop; % counter for number of strains

% Data for divisions, time has periodic boundary conditions
maxtime=round(initdivtime*growdisadvfact*1.25/tol); % cutoff when time resets to 0
timematS=zeros(limstrain,maxtime); % #S dividing for rows = strains, cols = time
timematW=zeros(limstrain,maxtime); % #W dividing for rows = strains, cols = time
timematS(1:startpop,round(initdivtime/tol))=maxstartpop/startpop; % init for Es
curtot=0; % counter for cell divisions

% Data saved
if startpop<=10
    datamat=zeros(numofrounds*2,startpop*5+1); % keeps # of strains, pop growth rate, pop trans. probs
else
    datamat=zeros(numofrounds*2,1*5+1); % keeps the top competitor
end
% Initialize
indW=8;indS=7;  % indices for storing data

for i1=1:numofrounds;
    % Growth in S
    done=0;curtime=1;curtot=0;
    N=Ns;
    while done<1;
        % Find those ready to divide
        totdiv=sum(timematS(1:strainnum,curtime))+sum(timematW(1:strainnum,curtime));
        if (totdiv+curtot>N)
            % All can divide
            timematS(1:strainnum,curtime)=timematS(1:strainnum,curtime)*(N-curtot)/totdiv;
            timematW(1:strainnum,curtime)=timematW(1:strainnum,curtime)*(N-curtot)/totdiv;
            done=1;
        end
        k=timematS(1:strainnum,curtime);
        for i2=1:strainnum
            if k(i2)>0
                ind=i2;
                % Dividing
                num2div=k(i2);
                % How many of the opposing type are created
                ptrans=strainmat(ind,3); % S-> W in Es
                num2other=num2div*ptrans;
                newtime=round(curtime+strainmat(ind,1)*growdisadvfact/tol);  % determine when they divide
                if newtime>maxtime;newtime=newtime-maxtime;end % periodic boundary conditions
                strainmat(ind,indW)=strainmat(ind,indW)+num2other; % increase W count
                timematW(ind,newtime)=timematW(ind,newtime)+num2other; % set next division time
                num2div=num2div-num2other; % number left to divide
                % Set next division time of current
                newtime=round(curtime+strainmat(ind,1)/tol);
                if newtime>maxtime;newtime=newtime-maxtime;end % periodic boundary conditions
                timematS(ind,newtime)=timematS(ind,newtime)+timematS(ind,curtime)+num2div; % set next division time
                strainmat(ind,indS)=strainmat(ind,indS)+num2div; % increase S count
            end
        end
        curtot=curtot+sum(k);
        % Growth of W types
        k=timematW(1:strainnum,curtime); % find those ready to divide
        for i2=1:strainnum
            if k(i2)>0
                ind=i2;
                % Dividing
                num2div=k(i2);
                % How many of the opposing type are created
                ptrans=strainmat(ind,4);
                num2other=num2div*ptrans;
                newtime=round(curtime+strainmat(ind,1)/tol);  % determine when they divide
                if newtime>maxtime;newtime=newtime-maxtime;end % periodic boundary conditions
                strainmat(ind,indS)=strainmat(ind,indS)+num2other; % increase S count
```

```
            timematS(ind,newtime)=timematS(ind,newtime)+num2other; % set next division time
            num2div=num2div-num2other; % number left to divide
            % Set next division time of current
            newtime=round(curtime+strainmat(ind,1)*growdisadvfact/tol);
            if newtime>maxtime;newtime=newtime-maxtime;end % periodic boundary conditions
            timematW(ind,newtime)=timematW(ind,newtime)+timematW(ind,curtime)+num2div; % set next division time
            strainmat(ind,indW)=strainmat(ind,indW)+num2div; % increase W count
        end
    end
    curtot=curtot+sum(k);
    curtime=curtime+1;
    if curtime>maxtime;curtime=curtime-maxtime;end % periodic boundary conditions
end

%%%%%%%%%%%%%%%%%%%%%%%%%%%%%%%%%%%%%%%%%%%%%%%%%%%%%%%%%%%%%%%%%%%%%%%%%%%%%%%%%%%%%%%%%%%%%%%%%%%%%%%%

% Transition back to W
timematS=zeros(limstrain,maxtime);
timematW=zeros(limstrain,maxtime);

% Pick which strains remain
totW=sum(strainmat(1:strainnum,indW)); % total amount of W
strainmat(1:strainnum,indW)=maxstartpop/totW*strainmat(1:strainnum,indW); % perfect filter
strainmat(1:strainnum,indS)=0; % perfect filter
for i9=1:strainnum
    timematW(i9,round(strainmat(i9,1)/tol))=strainmat(i9,indW);
end
% Display data and storage of results
datamat(i1*2-1,1)=i1/numofrounds;
if startpop<=10
    for i9=1:startpop
        datamat(i1*2-1,2+(i9-1)*5:i9*5+1)=[log10(strainmat(i9,7)+strainmat(i9,8)) log10(strainmat(i9,[3 4 5 6]))];
    end
else
    [ud,vd]=max(strainmat(1:strainnum,indW));
    datamat(i1*2-1,2:6)=[ud log10(strainmat(vd,3:6))];
end
% Reset parameters
done=0;curtime=1;N=Nw;
curtot=0; %number of cell divisions

%%%%%%%%%%%%%%%%%%%%%%%%%%%%%%%%%%%%%%%%%%%%%%%%%%%%%%%%%%%%%%%%%%%%%%%%%%%%%%%%%%%%%%%%%%%%%%%%%%%%%%%%

% Growth in W
while done<1;
    % Find those ready to divide
    totdiv=sum(timematW(1:strainnum,curtime))+sum(timematS(1:strainnum,curtime));
    if (totdiv+curtot>N)
        % All can divide
        timematW(1:strainnum,curtime)=timematW(1:strainnum,curtime)*(N-curtot)/totdiv;
        timematS(1:strainnum,curtime)=timematS(1:strainnum,curtime)*(N-curtot)/totdiv;
        done=1;
    end
    k=timematW(1:strainnum,curtime);
    for i2=1:strainnum
        if k(i2)>0
            ind=i2;
            % Dividing
            num2div=k(i2);
            % How many of the opposing type are created
            ptrans=strainmat(ind,6);
            num2other=num2div*ptrans;
            newtime=round(curtime+strainmat(ind,1)*growdisadvfact/tol);  % determine when they divide
            if newtime>maxtime;newtime=newtime-maxtime;end % periodic boundary conditions
            strainmat(ind,indS)=strainmat(ind,indS)+num2other; % increase S count
            timematS(ind,newtime)=timematS(ind,newtime)+num2other; % set next division time
            num2div=num2div-num2other; % number left to divide
            % Set next division time of current
            newtime=round(curtime+strainmat(ind,1)/tol);
```

```
            if newtime>maxtime;newtime=newtime-maxtime;end % periodic boundary conditions
            timematW(ind,newtime)=timematW(ind,newtime)+timematW(ind,curtime)+num2div; % set next division time
            strainmat(ind,indW)=strainmat(ind,indW)+num2div; % increase W count
        end
    end
    curtot=curtot+sum(k);

    % Growth of S types
    k=timematS(1:strainnum,curtime); % find those ready to divide
    for i2=1:strainnum
        if k(i2)>0
            ind=i2;
            % Dividing
            num2div=k(i2);
            % How many of the opposing type are created
            ptrans=strainmat(ind,5);
            num2other=num2div*ptrans;
            newtime=round(curtime+strainmat(ind,1)/tol);  % determine when they divide
            if newtime>maxtime;newtime=newtime-maxtime;end % periodic boundary conditions
            strainmat(ind,indW)=strainmat(ind,indW)+num2other; % increase W count
            timematW(ind,newtime)=timematW(ind,newtime)+num2other; % set next division time
            num2div=num2div-num2other; % number left to divide
            % Set next division time of current
            newtime=round(curtime+strainmat(ind,1)*growdisadvfact/tol);
            if newtime>maxtime;newtime=newtime-maxtime;end % periodic boundary conditions
            timematS(ind,newtime)=timematS(ind,newtime)+timematS(ind,curtime)+num2div; % set next division time
            strainmat(ind,indS)=strainmat(ind,indS)+num2div; % increase W count
        end
    end
    curtot=curtot+sum(k);
    curtime=curtime+1;
    if curtime>maxtime;curtime=curtime-maxtime;end % periodic boundary conditions
end

%%%%%%%%%%%%%%%%%%%%%%%%%%%%%%%%%%%%%%%%%%%%%%%%%%%%%%%%%%%%%%%%%%%%%%%%%%%%%%%%%%%%%%%%%%%%%%%%%%%%%
  % Transition back to S
  timematS=zeros(limstrain,maxtime);
  timematW=zeros(limstrain,maxtime);

  % Pick which strains remain
  totS=sum(strainmat(1:strainnum,indS)); % total amount of S
  strainmat(1:strainnum,indS)=maxstartpop/totS*strainmat(1:strainnum,indS); % perfect filter
  strainmat(1:strainnum,indW)=0; % perfect filter
  % Display data and storage of results
  datamat(i1*2,1)=i1/numofrounds;
  if startpop<=10
      for i9=1:startpop
          datamat(i1*2,2+(i9-1)*5:i9*5+1)=[log10(strainmat(i9,7)+strainmat(i9,8)) log10(strainmat(i9,[3 4 5 6]))];
      end
      [ud,vd]=max(datamat(i1*2,2:5:end)); % vd is index of dominant strain
      strainmat(1,3:8)=[strainmat(vd,3:6) maxstartpop/startpop 0];
  else
      [ud,vd]=max(strainmat(1:strainnum,indS));
      datamat(i1*2,2:6)=[ud log10(strainmat(vd,3:6))];
      strainmat(1,3:8)=[strainmat(vd,3:6) maxstartpop/startpop 0];
  end
  [i1/numofrounds log10(strainmat(1,3:6))] % displays the winner's properties
  for i9=2:startpop
   % create new competitors
      strainmat(i9,3:6)=strainmat(1,3:6);
      tmp=floor(4*rand(1,1));
      strainmat(i9,3+tmp)=10.^(-6*rand(1,1));
      strainmat(i9,7:8)=[maxstartpop/startpop 0];
  end
  timematS(1:startpop,round(initdivtime/tol))=maxstartpop/startpop; % initialize for division beginning in Es

%%%%%%%%%%%%%%%%%%%%%%%%%%%%%%%%%%%%%%%%%%%%%%%%%%%%%%%%%%%%%%%%%%%%%%%%%%%%%%%%%%%%%%%%%%%%%%%%%%%%%

end
```

```
 % Sample plot of winning transition probabilities
close all
plot(datamat(2:2:end,1)*numofrounds,10.^datamat(2:2:end,5),'o','Color',[.65 .65 .65],'LineWidth',3);
hold on;
plot(datamat(2:2:end,1)*numofrounds,10.^datamat(2:2:end,6),'o','Color',[.65 .65 .65],'LineWidth',3);
plot(datamat(2:2:end,1)*numofrounds,10.^datamat(2:2:end,3),'o','Color',[0 0 0],'LineWidth',3);
plot(datamat(2:2:end,1)*numofrounds,10.^datamat(2:2:end,4),'o','Color',[0 0 0],'LineWidth',3);
xlabel('Number of rounds','FontSize',14);
ylabel('Winning transition probability','FontSize',14);
axis square;
set(gca,'TickLength',[.025 .025],'yScale','log','LineWidth',3,'FontSize',14);
axis([0 numofrounds+1 10^-6.1 10^0 ]);
```
